# *catena*-Poly[[(pyrazine-2-carboxamide-κ*N* ^4^)copper(I)]-μ_3_-iodido]

**DOI:** 10.1107/S1600536814013695

**Published:** 2014-06-18

**Authors:** Lukáš Krivosudský, Erik Rakovský

**Affiliations:** a Comenius University, Faculty of Natural Sciences, Department of Inorganic Chemistry, Mlynská dolina CH2, 842 15 Bratislava, Slovak Republic

## Abstract

In the title metal–organic polymeric complex, [CuI(C_5_H_5_N_3_O)]_*n*_, the asymmetric unit is composed of one monomer unit of the polymer and one Cu^I^ atom linked to one iodide anion and one pyrazine-2-carboxamide mol­ecule. The Cu^I^ atom is in a distorted tetra­hedral coordination completed by one pyrazine N atom of the pyrazine-2-carboxamide ligand and three iodide anions. The polymeric structure adopts a well-known ladder-like motif of {CuNI_3_} tetra­hedra running in the *b*-axis direction. The mol­ecules of the organic ligand are connected *via* medium-to-strong N—H⋯O and N—H⋯N hydrogen bonds and weak π–π inter­actions [the distance between two parallel planes of the rings is 3.5476 (14) Å and the centroid–centroid contact is 4.080 (2) Å]. The title compound has a relatively high decomposition temperature (564 K) as a result of relatively strong covalent and non-covalent inter­actions inside and between the chains.

## Related literature   

For other Cu^I^ coordination polymers, see: Peng *et al.* (2006 ▶, 2010 ▶); Feng *et al.* (2006 ▶); Wu *et al.* (2005 ▶); Rath & Holt (1985 ▶); Rath *et al.* (1986 ▶). For complexes of pyrazine-2-carboxamide with other transition metals and studies of their biological activity, see: Somoskovi *et al.* (2004 ▶); Singh & Seth (1975 ▶); Azizov *et al.* (1978 ▶). For other Cu^I^ complexes of pyrazine-2-carboxamide, see: Munakata *et al.* (1997 ▶); Goher & Mautner (1999 ▶, 2000 ▶, 2001 ▶). For a description of the Cambridge Structural Database, see: Allen (2002 ▶). For non-covalent inter­actions, see: Bernstein *et al.* (1995 ▶); Bondi (1964 ▶); Janiak (2000 ▶); Jia *et al.* (2009 ▶); Wells (1975 ▶). For the riding constraints used in the refinement, see: Cooper *et al.* (2010 ▶).
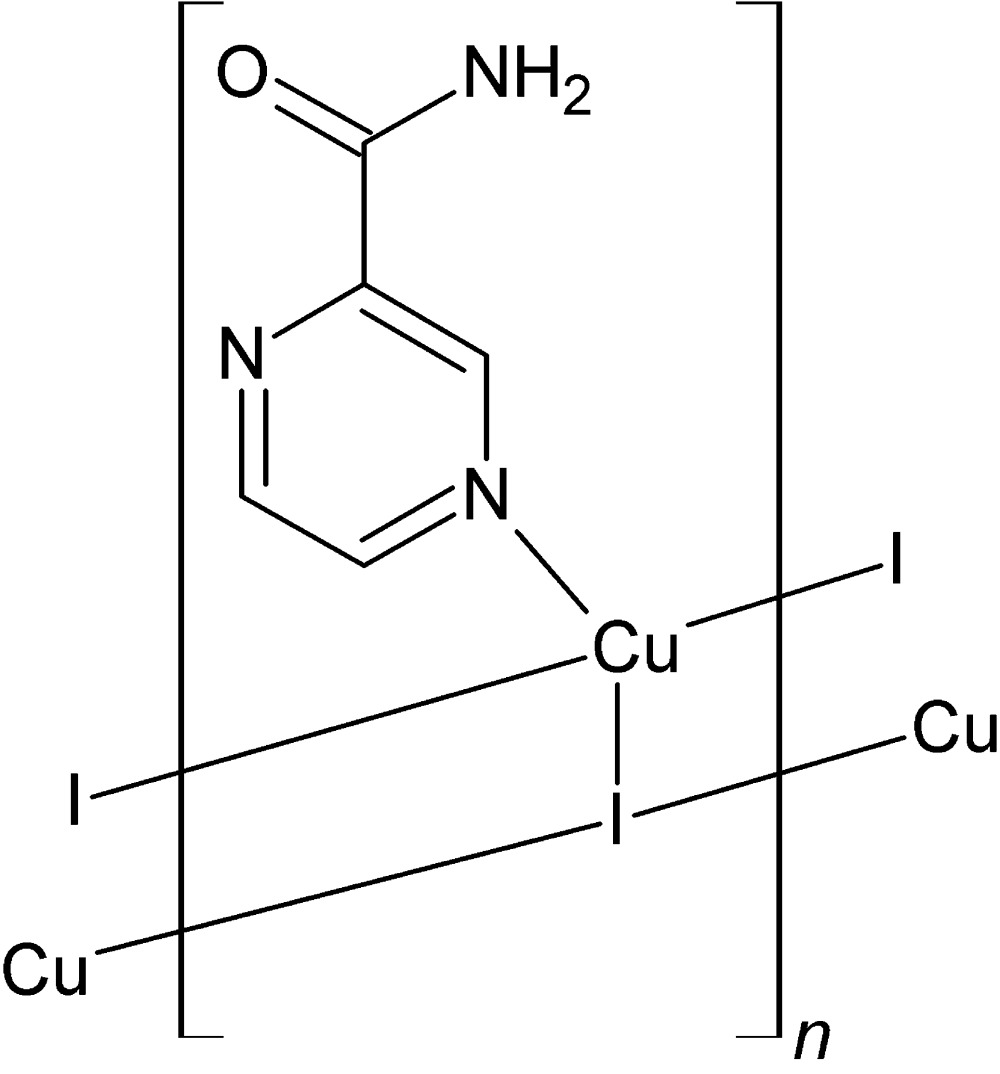


## Experimental   

### 

#### Crystal data  

[CuI(C_5_H_5_N_3_O)]
*M* *_r_* = 313.56
Monoclinic, 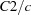

*a* = 29.5408 (7) Å
*b* = 4.0795 (1) Å
*c* = 14.3164 (3) Å
β = 111.712 (3)°
*V* = 1602.89 (7) Å^3^
*Z* = 8
Mo *K*α radiation
μ = 6.52 mm^−1^
*T* = 100 K
0.22 × 0.06 × 0.03 mm

#### Data collection  

Agilent SuperNova diffractometer
Absorption correction: multi-scan (*CrysAlis PRO*; Agilent, 2012 ▶) *T*_min_ = 0.562, *T*_max_ = 1.000
11538 measured reflections
2135 independent reflections
1339 reflections with *I* > 2σ(*I*)
*R*_int_ = 0.024

#### Refinement  

*R*[*F* ^2^ > 2σ(*F* ^2^)] = 0.021
*wR*(*F* ^2^) = 0.042
*S* = 1.00
1556 reflections
100 parameters
H-atom parameters constrained
Δρ_max_ = 0.96 e Å^−3^
Δρ_min_ = −0.87 e Å^−3^

### 

Data collection: *CrysAlis PRO* (Agilent, 2012 ▶); cell refinement: *CrysAlis PRO*; data reduction: *CrysAlis PRO*; program(s) used to solve structure: *SUPERFLIP* (Palatinus & Chapuis, 2007 ▶); program(s) used to refine structure: *CRYSTALS* (Betteridge *et al.*, 2003 ▶); molecular graphics: *DIAMOND* (Brandenburg, 1999 ▶), *Mercury* (Macrae *et al.*, 2006 ▶) and *ORTEP-3 for Windows* (Farrugia, 2012 ▶); software used to prepare material for publication: *CRYSTALS*, *PLATON* (Spek, 2009 ▶) and *publCIF* (Westrip, 2010 ▶).

## Supplementary Material

Crystal structure: contains datablock(s) I. DOI: 10.1107/S1600536814013695/vn2084sup1.cif

Structure factors: contains datablock(s) I. DOI: 10.1107/S1600536814013695/vn2084Isup2.hkl

Supporting information file. DOI: 10.1107/S1600536814013695/vn2084Isup3.mol

CCDC reference: 885829

Additional supporting information:  crystallographic information; 3D view; checkCIF report

## Figures and Tables

**Table 1:** Selected bond lengths (Å)

I1—Cu1^i^	2.6437 (5)
I1—Cu1^ii^	2.6310 (5)
I1—Cu1	2.6016 (5)
Cu1—N1	2.059 (3)
Cu1—Cu1^ii^	2.7974 (6)

Symmetry codes: (i) 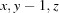; (ii) 
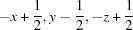
.

**Table 2:** Hydrogen-bond geometry (Å, °)

*D*—H⋯*A*	*D*—H	H⋯*A*	*D*⋯*A*	*D*—H⋯*A*
N3—H311⋯O1^iii^	0.84	2.05	2.883 (5)	170 (1)
N3—H312⋯N2^iv^	0.87	2.32	3.124 (5)	154 (1)

Symmetry codes: (iii) 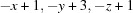; (iv) 
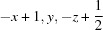
.

**Fig. 1.**
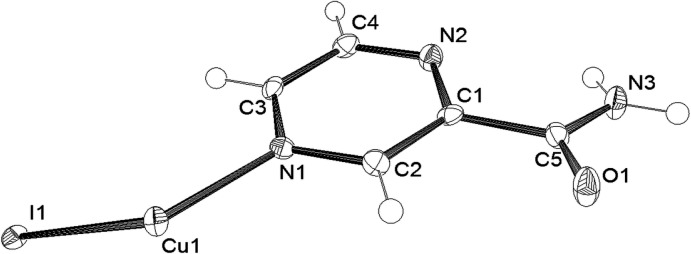
ADP representation of (I) with atom labeling scheme. The displacement ellipsoids are drawn at the 50% probability level, H atoms are drawn as spheres with arbitrary radii.

**Fig. 2.**
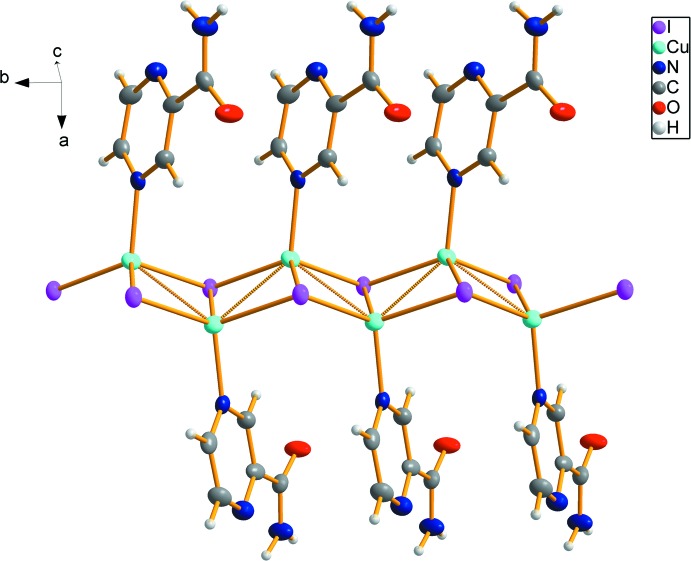
Polymeric structure of the title compound demonstrating the ladder–like motif of the {CuNI3} tetrahedrons. Displacement ellipsoids are drawn at the 80% probability level.

**Fig. 3.**
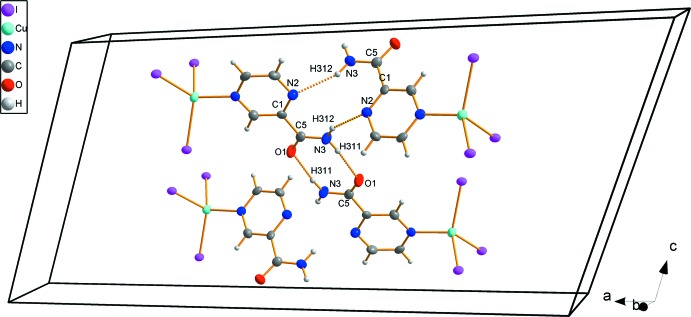
Eight- and ten-membered rings formed by hydrogen bonds in the crystal structure. Each polymer chain is attached to other two polymer chains.

**Fig. 4.**
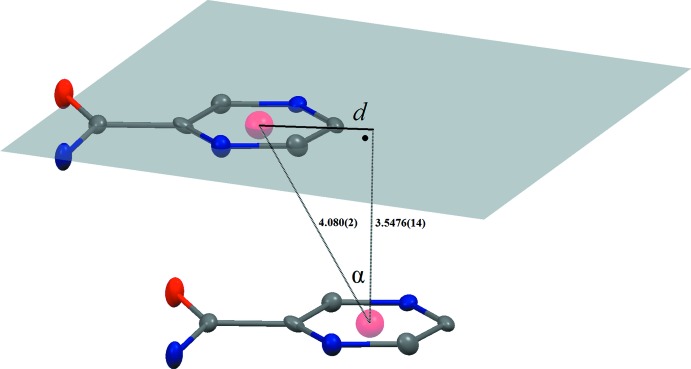
π–π stacking interaction in the crystal structure of (I). The angle α is the angle between the ring normal and centroid-centroid vector and d is the displacement between two rings (or centroids).

## supplementary crystallographic information

### S1. Comment

Copper(I) coordination polymers are well known for their photochemical and photophysical properties. There are several methods for the synthesis of such metal-organic frameworks (Peng *et al.*, 2010). One possible strategy is the use of copper(I) halide, especially iodide, which dissolves in concentrated aqueous solutions of iodides. After addition of an organic ligand polynuclear copper(I) complexes can be crystallized (Peng *et al.*, 2006; Feng *et al.*, 2006; Wu *et al.*, 2005; Rath & Holt, 1985; Rath *et al.*, 1986).

Pyrazine-2-carboxamide (usually pyrazinamide, in medical literature abbreviated as PZA) is a drug used for tuberculosis treatment in the last 60 years. Nowadays the possibility of increasing the activity of the drug by forming transition metal complexes is examinated widely (Somoskovi *et al.*, 2004). In addition to complexes with transition metals with potential biological effects, *e.g.* Mn(II), Ni(II), Fe(II), Zn(II), Cu(II) (Singh & Seth 1975; Azizov *et al.*, 1978; Goher & Mautner, 2000) the complexes with Cu(I) have also been prepared. They have been found to be water-insoluble stable compounds with and expected polymeric structure, as has been confirmed by X–ray structure analysis of six compounds with simplified formulas [Cu_2_(µ-PZA)_3_]*_n_*(ClO_4_)_2_.C_3_H_6_O, [Cu_2_(µ-PZA)_2_(µ-C_3_H_6_O)]*_n_*(BF_4_)_2_ (Munakata *et al.*, 1997); [Cu(µ-N_3_)PZA_2_]*_n_* (Goher & Mautner, 1999); [Cu(µ-I)PZA_2_]*_n_* (Goher & Mautner, 2000); [Cu(µ-CN)PZA_2_]*_n_* and [Cu(µ-SCN)PZA_2_]*_n_* (Goher & Mautner, 2001).

Goher & Mautner (2000) have succesfully prepared eleven new pyrazine-2-carboxamide complexes of Cu(I) including [CuI(pyrazine-2-carboxamide)]*_n_*, (I), but which was only obtained as a powder sample. We have successfully optimised the preparation process of (I) as well as other copper(I) complexes to obtain crystalline samples suitable for single crystal X-ray structure determination and the crystal structure of (I) is reported here.

As shown in Fig. 1, the asymmetric unit of (I) consists of one copper(I) cation bonded to one iodide anion and one pyrazine-2-carboxamide ligand. This unit is an elementary building block of catenary polymer as depicted in Fig. 2. The Cu^I^ is coordinated by one N atom from pyrazine-2-carboxamide ligand and three I^–^ anions to form a distorted tetrahedral coordination environment, where the Cu—N bond length is 2.059 (3) Å and the Cu—I bond lengths are 2.6016 (5), 2.6437 (5) and 2.6310 (5) Å. The Cu—N bond length is similar to the mean value of Cu—N bond lengths in above mentioned complexes (2.048 Å). There is a number of structures containing a similar ladder–like linkage of {CuNI_3_} tetrahedrons. In most cases one of the Cu—I bond lengths tends to be shorter then the other two, *e.g.* in the realated compound [Cu_2_(µ_3_-I)_2_(4-(4-carboxyphenyl)pyridine-N)_2_]*_n_* the Cu—I bond lengths are 2.631 (1), 2.662 (1), 2.667 (1) and 2.604 (1), 2.658 (1), 2.662 (1) Å (Jia *et al.*, 2009). The I—Cu—N bond angles are 105.07 (8), 114.64 (8) and 103.54 (8)°. The distance between two Cu^I^ centres 2.7974 (6) Å indicates that there there could be a weak metal—metal interaction as the sum of van der Waals radii of two copper atoms is 2.8 Å (Bondi, 1964).

The polymeric chains running in the *b* axis direction are connected via hydrogen bonds between the pyrazinamide ligands (Fig. 3). The amide groups of the ligands form typical dimers consisting of eight-membered rings with the graph set R_2_^2^(8) linked by a strong N–H···O hydrogen bond. The second hydrogen atom of the –NH_2_ group forms medium N–H···N hydrogen bond to the non-coordinating N atom of the pyrazine ring, thus forming a ten-membered ring with the graph set R_2_^2^(10) (Table 2.) (Bernstein *et al.* 1995).

Moreover, there is a weak π–π interaction between adjacent pyrazine rings in the polymer chain. The distance between two parallel planes of the rings is 3.5476 (14) Å and the centroid-centroid contact is 4.080 (2) Å (Fig. 4). The angle α between the ring normal and the centroid-centroid vector is 29.6 ° and the horizontal displacement of the rings *d* is 2.014 Å. It is well–known that often the ring planes are offset so that a ring atom lies almost over the center of the other ring and its hydrogen atom almost on the top of a carbon atom (Janiak, 2000). In the compound (I) this is not the case.

There are 9 compounds with the same ladder–like polymeric structure and pyrazine derivatives as the ligands in the CSD (Allen, 2002). The relevant parameters of the π–π interaction of these compounds compared to compound (I) are listed in Table 3. The entries DINQOG, GABHOH and ODOFOC correspond to the compounds with general formula [CuI(2-*sub*-pyrazine)]*_n_*, where *sub* is cyano–, iodo– and chloro– functional group respectively. More complicated substituents on the pyrazine ring obviously lead to an increasing α angle and displacement *d*. Interestingly, in the case of non–substituted pyrazine the two parameters are similar to those of compound (I).

Compound (I) is stable in air and insoluble in water and most solvents, it is only sparingly soluble in dimethylsulfoxide forming a pale orange solution. We performed a thermal decompostion analysis and we found that, surprisingly, the decomposition takes place at a quite high temperature (291 °C) starting with the release of pyrazine-2-carboxamide. The crystal structure analysis of (I) clearly elucidates that the organic molecule is well–anchored not only via covalent bonding to the copper(I) center but also via non-covalent interactions between pyrazine-2-carboxamide molecules. Thanks to the thermal stability and the photoluminiscence properties of (I) described in (Goher & Mautner, 2000) the compound is a possible candidate for the use as a photoactive material.

### S2. Experimental

All reactants except copper(I) iodide were obtained commercially and used without further purification. Copper(I) iodide was prepared as follows: Cu(NO_3_)_2_.3H_2_O (12.7 g, 52.5 mmol) was dissolved in 270 ml of distilled water and the solution was cooled until below room temperature. Na_2_S_2_O_3_.5H_2_O (13.03 g, 52.5 mmol) and KI (9.6 g, 57.8 mmol, 10% excess) were dissolved in 20 ml of distilled water and added slowly into the solution of copper(II) nitrate. The resulting mixture was heated for 20 minutes, cooled to room temperature and filtered. The product was washed with 50 ml of distilled water, 20 ml of ethanol and 20 ml of acetone. *Yield*: 9.4 g, 94%.

Synthesis of the [Cu(*µ*_3_-I)(C_5_H_5_N_3_O)]*_n_*(I): CuI (1 g, 5.25 mmol) was dissolved in 50 ml of 3.5 *M* solution of KI. Pyrazine-2-carboxamide (0.6464 g, 5.25 mmol) was added and the solution was boiled. The almost clear solution was filtered quickly and boiled again. The solution was slowly cooled to room temperature and placed in a refrigerator for 20 minutes. Fine red needle crystals of (I) were filtered, washed with distilled water (50 ml), ethanol (10 ml) and diethylether (10 ml). *Yield* 1.33 g, 80.8%.

Copper was determined gravimetrically as CuO. C, H and N were estimated on a CHN analyser Vario MICRO cube. Analysis calculated for C_5_H_5_N_3_O_1_I_1_Cu_1_ (found): C 19.15 (19.52), H 1.61 (1.43), N 13.40 (13.68), Cu 20.27 (19.91).

FT—IR spectra were obtained using an FT—IR Nicolet Magna 750 spectrophotometer. The IR spectrum of prepared compound exhibits characteristic bands of pyrazine-2-carboxamide: 3374(*s*) - ν_as_(NH_2_), 3161(*s*) - ν_s_(NH_2_), 1612(*s*) - ν(NH_2_), 1101(*w*) - δ(NH_2_), 1376(*s*) - amide, 1706(*s*) - ν(C=O), 529(*m*) - δ(OCN) (Goher & Mautner, 2000).

The thermal decomposition of the substance has been studied under controlled heating at a rate of 5°C.min^–1^ to 800°C in air using a derivatograph Q-1500 D device. In the first step of thermal decomposition pyrazine-2-carboxamide is released (291 °C, exothermal process) and copper(I) iodide is formed. At 381°C γ-CuI with the sphalerite structure is exothermically transformed to β-CuI adopting wurtzite structure (Wells, 1975). Finally copper(I) iodide is oxidized to copper(II) oxide and molecular iodine is released at 487°C.

### S3. Refinement

Crystal data, data collection and structure refinement details are summarized in Table 1. All non-H atoms were refined anisotropically as free atoms. The H atoms were all located in a difference map, but those attached to carbon atoms were repositioned geometrically. The H atoms were initially refined with soft restraints on the bond lengths and angles to regularize their geometry (C—H in the range 0.93–0.98, N—H in the range 0.86–0.89) and *U*_iso_(H) (in the range 1.2–1.5 times *U*_eq_ of the parent atom), after which the positions were refined with riding constraints (Cooper *et al.*, 2010).

### Crystal data

**Table:**

[CuI(C_5_H_5_N_3_O)]	*F*(000) = 1168
*M**_r_* = 313.56	*D*_x_ = 2.599 Mg m^−^^3^
Monoclinic, *C*2/*c*	Mo *K*α radiation, λ = 0.71073 Å
Hall symbol: -C 2yc	Cell parameters from 6637 reflections
*a* = 29.5408 (7) Å	θ = 2–30°
*b* = 4.0795 (1) Å	µ = 6.52 mm^−^^1^
*c* = 14.3164 (3) Å	*T* = 100 K
β = 111.712 (3)°	Needle, red
*V* = 1602.89 (7) Å^3^	0.22 × 0.06 × 0.03 mm
*Z* = 8	

### Data collection

**Table:**

Agilent SuperNova diffractometer	2135 independent reflections
Radiation source: SuperNova (Mo) X-ray Source	1339 reflections with *I* > 2σ(*I*)
Mirror monochromator	*R*_int_ = 0.024
Detector resolution: 10.3801 pixels mm^-1^	θ_max_ = 29.2°, θ_min_ = 2.9°
ω scans	*h* = −40→37
Absorption correction: multi-scan (*CrysAlis PRO*; Agilent, 2012)	*k* = −5→5
*T*_min_ = 0.562, *T*_max_ = 1.000	*l* = −19→14
11538 measured reflections	

### Refinement

**Table:**

Refinement on *F*^2^	Primary atom site location: iterative
Least-squares matrix: full	Secondary atom site location: difference Fourier map
*R*[*F*^2^ > 2σ(*F*^2^)] = 0.021	Hydrogen site location: difference Fourier map
*wR*(*F*^2^) = 0.042	H-atom parameters constrained
*S* = 1.00	Method = Modified Sheldrick *w* = 1/[σ^2^(*F*^2^) + 6.36*P*] where *P* = (max(*F*_o_^2^,0) + 2*F*_c_^2^)/3
1556 reflections	(Δ/σ)_max_ = 0.001
100 parameters	Δρ_max_ = 0.96 e Å^−^^3^
0 restraints	Δρ_min_ = −0.87 e Å^−^^3^

### Special details

**Table:**

Experimental. Absorption correction: *CrysAlis PRO* (Agilent, 2012) Empirical absorption correction using spherical harmonics, implemented in SCALE3 ABSPACK scaling algorithm.

### Fractional atomic coordinates and isotropic or equivalent isotropic displacement parameters (Å^2^)

**Table:**

	*x*	*y*	*z*	*U*_iso_*/*U*_eq_	
I1	0.220190 (8)	0.31394 (6)	0.101635 (17)	0.0111	
Cu1	0.268365 (16)	0.80730 (12)	0.20718 (3)	0.0128	
N1	0.33830 (11)	0.8564 (7)	0.2104 (2)	0.0107	
C2	0.37134 (13)	1.0182 (9)	0.2869 (3)	0.0127	
C1	0.41814 (13)	1.0748 (9)	0.2896 (3)	0.0111	
N2	0.43237 (11)	0.9779 (7)	0.2156 (2)	0.0142	
C4	0.39912 (14)	0.8216 (10)	0.1390 (3)	0.0156	
C3	0.35231 (13)	0.7580 (8)	0.1366 (3)	0.0117	
C5	0.45324 (13)	1.2545 (8)	0.3789 (3)	0.0123	
O1	0.43762 (9)	1.3879 (7)	0.4387 (2)	0.0187	
N3	0.49934 (11)	1.2586 (7)	0.3874 (2)	0.0171	
H21	0.3625	1.0951	0.3397	0.0157*	
H41	0.4076	0.7545	0.0853	0.0177*	
H31	0.3305	0.6449	0.0825	0.0152*	
H311	0.5204	1.3547	0.4365	0.0212*	
H312	0.5094	1.1527	0.3464	0.0198*	

### Atomic displacement parameters (Å^2^)

**Table:**

	*U*^11^	*U*^22^	*U*^33^	*U*^12^	*U*^13^	*U*^23^
I1	0.01335 (12)	0.00921 (12)	0.00965 (12)	−0.00022 (11)	0.00295 (9)	−0.00083 (11)
Cu1	0.0102 (2)	0.0136 (2)	0.0134 (2)	−0.0017 (2)	0.00299 (18)	−0.0011 (2)
N1	0.0123 (16)	0.0075 (16)	0.0123 (16)	−0.0001 (13)	0.0044 (13)	0.0031 (13)
C2	0.0143 (19)	0.012 (2)	0.0126 (19)	0.0009 (16)	0.0056 (16)	0.0001 (16)
C1	0.0106 (18)	0.0130 (18)	0.0091 (18)	0.0028 (15)	0.0029 (15)	0.0056 (15)
N2	0.0131 (16)	0.0161 (18)	0.0129 (16)	−0.0003 (14)	0.0042 (14)	−0.0014 (14)
C4	0.018 (2)	0.0193 (19)	0.0108 (18)	0.0037 (18)	0.0071 (16)	0.0004 (18)
C3	0.0113 (18)	0.009 (2)	0.0102 (18)	−0.0003 (14)	−0.0013 (15)	0.0010 (14)
C5	0.0119 (19)	0.011 (2)	0.0125 (19)	−0.0009 (14)	0.0023 (15)	0.0004 (15)
O1	0.0128 (14)	0.0252 (17)	0.0194 (15)	−0.0049 (12)	0.0074 (12)	−0.0105 (12)
N3	0.0142 (17)	0.021 (2)	0.0162 (17)	−0.0036 (14)	0.0056 (14)	−0.0092 (14)

### Geometric parameters (Å, º)

**Table:**

I1—Cu1^i^	2.6437 (5)	C1—N2	1.336 (5)
I1—Cu1^ii^	2.6310 (5)	C1—C5	1.505 (5)
I1—Cu1	2.6016 (5)	N2—C4	1.333 (5)
Cu1—N1	2.059 (3)	C4—C3	1.394 (5)
Cu1—Cu1^ii^	2.7974 (6)	C4—H41	0.933
Cu1—Cu1^iii^	2.7974 (6)	C3—H31	0.927
N1—C2	1.341 (5)	C5—O1	1.240 (4)
N1—C3	1.331 (5)	C5—N3	1.321 (5)
C2—C1	1.388 (5)	N3—H311	0.843
C2—H21	0.939	N3—H312	0.866
			
Cu1^i^—I1—Cu1^ii^	64.057 (14)	C2—N1—C3	116.8 (3)
Cu1^i^—I1—Cu1	102.106 (17)	N1—C2—C1	121.4 (3)
Cu1^ii^—I1—Cu1	64.633 (14)	N1—C2—H21	119.2
Cu1^ii^—Cu1—Cu1^iii^	93.63 (3)	C1—C2—H21	119.4
Cu1^ii^—Cu1—I1^iv^	127.34 (3)	C2—C1—N2	122.1 (3)
Cu1^iii^—Cu1—I1^iv^	57.750 (12)	C2—C1—C5	118.1 (3)
Cu1^ii^—Cu1—I1^iii^	58.193 (19)	N2—C1—C5	119.8 (3)
Cu1^iii^—Cu1—I1^iii^	57.174 (19)	C1—N2—C4	116.2 (3)
I1^iv^—Cu1—I1^iii^	114.921 (19)	N2—C4—C3	122.1 (3)
Cu1^ii^—Cu1—I1	58.193 (12)	N2—C4—H41	118.5
Cu1^iii^—Cu1—I1	126.95 (3)	C3—C4—H41	119.3
I1^iv^—Cu1—I1	102.106 (17)	C4—C3—N1	121.4 (3)
I1^iii^—Cu1—I1	116.38 (2)	C4—C3—H31	119.5
Cu1^ii^—Cu1—N1	127.58 (8)	N1—C3—H31	119.2
Cu1^iii^—Cu1—N1	117.90 (8)	C1—C5—O1	119.0 (3)
I1^iv^—Cu1—N1	105.07 (8)	C1—C5—N3	116.6 (3)
I1^iii^—Cu1—N1	103.54 (8)	O1—C5—N3	124.4 (3)
I1—Cu1—N1	114.64 (8)	C5—N3—H311	120.0
Cu1—N1—C2	119.1 (2)	C5—N3—H312	122.1
Cu1—N1—C3	123.9 (2)	H311—N3—H312	117.7

Symmetry codes: (i) *x*, *y*−1, *z*; (ii) −*x*+1/2, *y*−1/2, −*z*+1/2; (iii) −*x*+1/2, *y*+1/2, −*z*+1/2; (iv) *x*, *y*+1, *z*.

### Hydrogen-bond geometry (Å, º)

**Table:**

*D*—H···*A*	*D*—H	H···*A*	*D*···*A*	*D*—H···*A*
N3—H311···O1^v^	0.84	2.05	2.883 (5)	170 (1)
N3—H312···N2^vi^	0.87	2.32	3.124 (5)	154 (1)

Symmetry codes: (v) −*x*+1, −*y*+3, −*z*+1; (vi) −*x*+1, *y*, −*z*+1/2.
